# Plasma fetuin-A/α2-HS-glycoprotein correlates negatively with inflammatory cytokines, chemokines and activation biomarkers in individuals with type-2 diabetes

**DOI:** 10.1186/s12865-016-0171-y

**Published:** 2016-09-26

**Authors:** Sardar Sindhu, Nadeem Akhter, Steve Shenouda, Ajit Wilson, Rasheed Ahmad

**Affiliations:** Immunology & Innovative Cell Therapy Unit, Dasman Diabetes Institute (DDI), P.O. Box 1180, Dasman, 15462 Kuwait

**Keywords:** Fetuin-A, α2-HS-glycoprotein, Type-2 diabetes, Proinflammatory cytokines, Chemokines, Metabolic inflammation

## Abstract

**Background:**

Fetuin-A/AHSH is a novel hepatokine that acts as a vascular calcification inhibitor and as an endogenous TLR-4 ligand. Fetuin-A may act as a positive or negative acute phase protein (APP) in disease conditions. The relationship between circulatory fetuin-A and inflammatory biomarkers in type-2 diabetes (T2D) remains controversial. Therefore, the purpose of this study was to determine the plasma fetuin-A levels in 53 T2D (BMI = 29.7 ± 4.5 kg/m^2^) and 72 non-diabetic individuals (BMI = 28.2 ± 5.8 kg/m^2^) using premixed 38-plex MAP human cytokine/chemokine magnetic bead immunoassays and the data (mean ± SEM) were statistically analyzed to determine Pearson’s correlation (r) between fetuin-A and detected analytes; *P*-values ≤0.05 were considered significant.

**Results:**

The data show that plasma fetuin-A levels were comparable in both groups (*P* = 0.27) and in T2D individuals, fetuin-A associated negatively (*P* ≤ 0.05) with a large number of proinflammatory cytokines/chemokines and activation biomarkers including TNF-α, IFN-α2, IFN-γ, IL-1α, IL-1β, IL-1RA, IL-3, IL-4, IL-7, IL-9, IL-12p40/p70, IL-15, CCL-2, CCL-4, CCL-11, CCL-22, CXCL-8, CX3CL-1, EFF-2, EGF, G-CSF, GM-CSF, GRO, sCD40L, and VEGF. In non-diabetics, fetuin-A also correlated positively with certain T_H_2 cytokines (IL-5, IL-13) and chemokines (CCL-3, CCL-5, CCL-7). Notably, in vitro fetuin-A production was significantly suppressed in HepG2 cells treated with TNF-α, IL-1β, and IFN-γ which supported the clinical findings of a negative association between fetuin A and inflammatory mediators.

**Conclusions:**

The negative association between circulatory fetuin-A and systemic inflammatory mediators in T2D patients suggests that plasma fetuin-A may have predictive significance as a negative APP in metabolic disease.

**Electronic supplementary material:**

The online version of this article (doi:10.1186/s12865-016-0171-y) contains supplementary material, which is available to authorized users.

## Background

Fetuin-A, also known as α-2 Heremans-Schmid glycoprotein (AHSG), is a member of cystatin superfamily of protease inhibitors and is a major human secretory protein of hepatic origin with multiple normal biological as well as pathological functions including inhibition of vascular calcification, bone metabolism regulation, control of protease activity, insulin resistance, keratinocyte migration, and breast tumor cell proliferative signaling [1]. It is a highly expressed glycoprotein in various fetal tissues whereas it is mainly expressed by the liver in adults [2]. Fetuin-A acts as an endogenous ligand for the innate immune Toll-like receptor (TLR)-4 and it also binds to β-subunit of insulin receptor to inactivate the insulin receptor tyrosine kinase for modulating insulin signaling [3].

The emerging evidence weighs in with both the pro- and anti-inflammatory attributes of fetuin-A. As a positive acute phase protein (APP), fetuin-A was reported to induce the expression of inflammatory cytokines in adipocytes and macrophages and was regarded as an inflammatory marker [4]. On the other hand, as a negative APP, fetuin-A glycoprotein was found to play a protective or anti-inflammatory role in various disease conditions such as infection, sepsis, endotoxemia, trauma, cerebral ischemic injury, autoimmune disorders, and Alzheimer’s disease [3].

Type-2 diabetes (T2D) is marked by a state of chronic low-grade inflammation called metabolic inflammation and the predictive value of circulatory fetuin-A as a positive or a negative APP in T2D-associated metabolic inflammation remains controversial [5, 6]. Therefore, the aim of the study was to find the relationship between plasma fetuin-A and signature inflammatory mediators and biomarkers in T2D patients. Herein, we report that plasma fetuin-A levels correlated negatively with inflammatory cytokines/chemokines and activation biomarkers in T2D individuals, suggesting its predictive importance as a negative APP in metabolic inflammation.

## Methods

### Study population and clinical assays

This study included 53 T2D patients (aged 23–75 years) including 11 lean with body mass index (BMI) of 23.9 ± 1.6 kg/m^2^, 18 overweight (BMI = 27.7 ± 1.5 kg/m^2^), and 24 obese individuals (BMI = 33.9 ± 2.5 kg/m^2^) and 72 non-diabetic individuals (aged 21–66 years) including 24 lean (BMI = 22.4 ± 1.9 kg/m^2^), 23 overweight (BMI = 27.1 ± 1.5 kg/m^2^), and 25 obese individuals (BMI = 34.8 ± 3.6 kg/m^2^), recruited through clinics of Dasman Diabetes Institute, Kuwait following study approval by the institutional ethics committee. Those of age <18 years or with serious lung, kidney, liver, or cardiovascular disease, hematologic or immune disorders, type-1 diabetes, pregnancy, or malignancy were excluded. Plasma samples were analyzed for fasting plasma glucose, glycated hemoglobin (HbA1c), total cholesterol, high- and low-density lipoproteins, triglycerides, and adiponectin using commercial kits following instructions as recommended by the manufacturers. Glucose and lipid profiles were measured using Siemens dimension RXL chemistry analyzer (Diamond Diagnostics, Holliston, MA, USA) and HbA1c was measured using Variant device (BioRad, Hercules, CA, USA). T2D diagnosis was based on results of (i) fasting plasma glucose, (ii) oral glucose tolerance test (OGTT), and (iii) HbA1c test. Fasting blood glucose levels of ≥126 mg/dL (≥7 mmol/L), 2 h-OGTT values of >200 mg/dL (11.1 mmol/L), and/or HbA1C levels of ≥6.5 % on two separate tests were diagnosed by physician as T2D. Regarding anthropometric and physical data, height and weight were measured while barefoot, waist circumference was measured and the waist-to-hip ratio was calculated. BMI was calculated as follows: body weight (kg)/height (m^2^). An average of three blood pressure readings (Omron HEM-907XL digital automatic sphygmomanometer, Omron Healthcare Inc. IL, USA), taken after 5–10 min rest for each, was obtained. The whole body composition including body fat percentage, soft lean mass and total body water was assessed (IOI 353 Body Composition Analyzer, Jawon Medical, South Korea). Patients’ clinico-demographic data are summarized in Table 1.

**Table 1:** Patients’ characteristics and clinical data

Parameter	Diabetic			Non-diabetic		
	Lean	Overweight	Obese	Lean	Overweight	Obese
Total number (N)	11	18	24	24	23	25
Male (N)	7	9	14	10	7	11
Female (N)	4	9	10	14	16	14
Age (Yrs.)	28–58	36–61	23–75	21–61	23–60	28–66
Body mass index (kg/m^2^)	23.9 ± 1.6	27.7 ± 1.5	33.9 ± 2.5	22.4 ± 1.9	27.1 ± 1.5	34.8 ± 3.6
Body fat percentage	28.9 ± 4.9	33.5 ± 5.9	36.9 ± 5.5	31.3 ± 5.1	33.8 ± 4.6	39.1 ± 5.0
Fasting plasma glucose (mmol/L)	7.9 ± 2.6	7.5 ± 2.8	8.7 ± 2.6	5.1 ± 0.8	5.2 ± 0.4	5.6 ± 0.9
Glycated hemoglobin (HbA1c) (%)	7.4 ± 1.8	6.9 ± 1.3	8.7 ± 1.9	5.5 ± 0.5	5.6 ± 0.4	6.0 ± 1.5
Total cholesterol (mmol/L)	5.1 ± 1.3	5.1 ± 2.0	5.2 ± 1.2	4.9 ± 1.1	5.2 ± 0.9	3.6 ± 0.9
High-density lipoprotein (mmol/L)	1.4 ± 1.0	1.2 ± 0.5	1.2 ± 0.3	1.4 ± 0.4	1.4 ± 0.5	1.2 ± 0.3
Low-density lipoprotein (mmol/L)	3.2 ± 0.7	3.3 ± 1.7	3.2 ± 1.0	3.2 ± 0.8	3.2 ± 0.8	3.5 ± 0.9
Triglycerides (mmol/L)	1.3 ± 0.8	1.6 ± 1.4	2.0 ± 1.6	0.8 ± 0.3	1.1 ± 0.6	1.3 ± 0.6
Hypertension (N)	1	12	12	0	3	7
Hyperlipidemia (N)	1	1	3	3	0	2
Therapy	Lipitor Insulin Aldomet	Metformin Lipitor Novorapid Concor	Metformin Diamicron NovoRapid Lipitor Concor	Lipitor	Lipitor Concor	Lipitor Aldomet Eltoxin

### Plasma analytes

The circulatory levels of a panel of 38 analytes including proinflammatory cytokines, chemokines, and activation/growth biomarkers were assessed using premixed 38-plex MAP human cytokine/chemokine magnetic bead (HCYTMAG-60 K-PX38, EMD Millipore Corp, USA) multiplex immunoassay. Data were acquired using Luminex xMAP analyzer (Luminex 100/200 Milliplex Analyzer, Luminex Corp. USA) following the manufacturer’s instructions while a digital data output processor and Milliplex analytical software were used to determine mean fluorescence intensity (MFI) and concentrations (pg/mL) of analytes. The data were statistically analyzed using unpaired *t*-test and the linear dependence between two variables was assessed by Pearson’s correlation coefficient (r) using GraphPad Prism software (version 6.05; San Diego, CA, USA). All *P*-values ≤0.05 were considered as statistical significant.

### Cell culture and measurement of in vitro fetuin-A production

Human hepatocellular carcinoma cell line HepG2 cells were cultured at 37 °C with 5 % CO_2_ in high-glucose DMEM medium containing 10 % (v/v) fetal bovine serum, 100 units/mL penicillin, and 100 μg/mL streptomycin in 6-well plates at a density of 0.25 × 10^6^ cells/mL until about 70 % confluence and old medium was replaced with fresh culture medium. Cell monolayers were treated with rhTNF-α (50 ng/mL), rhIL-1β (10 ng/mL), rhIFN-γ (50 ng/mL), rhMCP-1 (40 ng/mL), and rhIL-6 (100 ng/mL) and incubated at 37 °C for 24 h. In time course experiments, HepG2 cell monolayers were treated with rhIFN-γ (50 ng/mL), rhTNF-α (50 ng/mL), rhIL-4 (20 ng/mL), and rhIL-10 (30 ng/mL) and cell cultures were incubated at 37 °C for 1, 6, 12, 24, and 48 h. Cell supernatants were collected, clarified by centrifugation, aliquoted, and stored at −80° until use. Fetuin-A levels were measured in cell supernatants using sandwich high-sensitivity ELISA (Sensitivity: <10 pg/mL; Human fetuin-A PicoKine™ ELISA kit, Boster Biological Technology, USA) following the manufacturer’s instructions. Briefly, after adding samples (100 μL) and standards (100 μL), plates were incubated at 37 °C for 90 min. Then, without washing, biotinylated anti-human fetuin-A antibody (1:100 diluted; 130 μL/well) was added and incubated at 37 °C for 60 min. Plates were washed 3 times with 0.01 M Tris-buffered saline (TBS), then Avidin-Biotin-Peroxidase Complex (ABC) was added (1:100 diluted; 130 μL/well) and incubated at 37 °C for 30 min. Plates were washed 5 times with TBS buffer and after adding HRP substrate 3,3',5,5'-Tetramethylbenzidine (TMB color developing agent), plates were incubated at 37 °C in dark for 20–25 min. Color development was stopped by adding TMB stop solution (100 μL/well) and absorbance/optical density (OD) was read at 450 nm wavelength. There was no cross reactivity detected against bovine or fetal calf fetuin-A in control wells containing only the culture media.

## Results

### Plasma fetuin-A associates negatively with the inflammatory cytokines/chemokines and activation biomarkers

The oxidative stress and inflammatory responses induced in obesity and T2D are associated with upregulated circulatory levels of various proinflammatory cytokines, chemokines, and stimulatory factors that may act in autocrine/paracrine manners to establish a sustained, low-grade chronic inflammation, called metabolic inflammation, over time. We asked whether plasma fetuin-A was related as a positive or negative APP in metabolic inflammation. This led us to perform multiplex analysis of a wide array of these proinflammatory cytokine/chemokines and related activation biomarkers in plasma samples of diabetic and non-diabetic individuals including determination of the circulatory fetuin-A levels. To this end, we found that although plasma fetuin-A levels were comparable between two groups (*P* = 0.27), T2D individuals had significantly higher plasma concentrations (*P* ≤ 0.05) of interferon (IFN)-α2, interleukin (IL)-1α, IL-1β, IL-4, IL-7, IL-12p40, tumor necrosis factor (TNF)-α, C-C motif ligand (CCL)-2/macrophage chemoattractant protein (MCP)-1, CCL-5/regulated on activation, normal T cell expressed and secreted (RANTES), CCL-11/eotaxin-1, C-X3-C motif ligand (CX3CL)-1/fractalkine, epidermal growth factor (EGF), elongation factor F (EFF)-2, granulocyte-colony stimulating factor (G-CSF), and soluble CD40 ligand (sCD40L) as compared with non-diabetic individuals (data are summarized in Table 2). Next, plasma fetuin-A levels in T2D patients were associated negatively (*P* ≤ 0.05) with proinflammatory cytokines/chemokines and activation biomarkers including IFN-α2, IFN-γ, IL-1α, IL-1β, IL-1RA, IL-4, IL-7, IL-9, IL-12p40, IL-12p70, IL-15, IL-17A, TNF-α, CCL-2/MCP-1, CCL-4/macrophage inflammatory protein (MIP)-1β, CCL-11/eotaxin-1, CCL-22/macrophage-derived chemokine (MDC), C-X-C motif ligand (CXCL)-8/IL-8, CX3CL-1/Fractalkine, growth-related oncogene (GRO), EGF, EFF-2, G-CSF, granulocyte-macrophage colony stimulating factor (GM-CSF), vascular endothelial growth factor (VEGF), and sCD40L (Table 3). In non-diabetic individuals, plasma fetuin-A correlated negatively (*P* ≤ 0.05) with IL-1β, IL-3, IL-7, IL-9, IL-15, TNF-α, CCL-2/MCP-1, CCL-22/MDC, CX3CL-1/Fractalkine, CXCL-10/IP-10, GRO, EFF-2, sCD40L, and VEGF. While, in this cohort, plasma fetuin-A also correlated positively (*P* ≤ 0.05) with: (a) certain allergic mediators and T_H_2 cytokines such as IL-5 and IL-13; (b) a multifunctional T_H_1 cytokine called lymphotoxin (LT)-α/TNF-β; and (c) certain chemokines such as CCL-3/MIP-1α, CCL-5/RANTES, and CCL-7/MCP-3 (Table 3). Both in T2D and non-diabetic individuals, no association (*P* > 0.05) was found between plasma fetuin-A and circulatory IL-6, IL-10, adiponectin, FMS-like tyrosine kinase 3 ligand (FLT-3 L) and transforming growth factor (TGF)-α.

**Table 2:** Plasma analytes (pg/mL) in type-2 diabetic and non-diabetic individuals

Analyte type	Diabetic		Non-diabetic		*P*
	Mean (Samples)	SEM	Mean (Samples)	SEM	
Cytokines					
IFN-α2	73.56 (*n* = 52)	8.58	50.99 (*n* = 56)	7.22	0.05*
IFN-γ	7.11 (*n* = 49)	0.83	10.96 (*n* = 66)	1.72	0.07
IL-1α	58.91 (*n* = 46)	8.48	33.32 (*n* = 56)	5.84	0.01*
IL-1β	3.42 (*n* = 46)	0.45	2.04 (*n* = 58)	0.20	0.004**
IL-1RA	18.92 (*n* = 52)	2.84	23.72 (*n* = 63)	5.88	0.49
IL-3	10.10 (*n* = 24)	2.34	1.45 (*n* = 14)	0.26	0.10
IL-4	14.67 (*n* = 49)	2.47	7.35 (*n* = 60)	0.83	0.003**
IL-5	2.12 (*n* = 20	0.37	10.22 (*n* = 19)	2.67	0.004**
IL-6	3.89 (*n* = 29)	0.74	4.26 (*n* = 33)	0.79	0.74
IL-7	6.80 (*n* = 51)	0.93	4.51 (*n* = 61)	0.52	0.03*
IL-9	2.34 (*n* = 18)	0.52	1.81 (*n* = 9)	0.26	0.50
IL-10	4.88 (*n* = 40)	0.51	6.86 (*n* = 36)	3.60	0.57
IL-12p40	31.21 (*n* = 39)	4.59	16.92 (*n* = 36)	2.05	0.007**
IL-12p70	7.86 (*n* = 49)	1.10	6.13 (*n* = 59)	0.63	0.15
IL-13	10.10 (*n* = 26)	2.34	56.15 (*n* = 20)	16.72	0.003**
IL-15	3.37 (*n* = 41)	0.54	2.36 (*n* = 34)	0.29	0.13
IL-17A	4.14 (*n* = 40)	0.52	4.40 (*n* = 50)	0.81	0.80
TNF-α	9.59 (*n* = 50)	0.78	6.54 (*n* = 72)	0.42	<0.001***
TNF-β/LT-α	5.66 (*n* = 20)	1.08	79.58 (*n* = 16)	31.73	0.01*
Chemokines					
CCL-2/MCP-1	209.30 (*n* = 53)	17.28	158.70 (*n* = 72)	8.99	0.006**
CCL-3/MIP1-α	7.09 (*n* = 48)	0.48	21.76 (*n* = 53)	13.24	0.29
CCL-4/MIP1-β	16.09 (*n* = 53)	1.40	17.03 (*n* = 71)	1.94	0.72
CCL-5/RANTES	87146.0 (*n* = 48)	7136.0	57930.0 (*n* = 63)	4280.0	<0.001***
CCL-7/MCP-3	10.21 (*n* = 10)	1.96	75.48 (*n* = 14)	23.24	0.03*
CCL-11/Eotaxin-1	67.94 (*n* = 53)	3.62	52.07 (*n* = 72)	2.18	<0.001***
CCL-22/MDC	385.60 (*n* = 53)	22.73	441.80 (*n* = 72)	37.89	0.25
CX3CL-1/Fractalkine	199.80 (*n* = 53)	17.76	149.80 (*n* = 70)	8.50	0.007**
CXCL-8/IL-8	4.60 (*n* = 47)	0.78	6.82 (*n* = 52)	1.44	0.19
CXCL-10/IP-10	278.70 (*n* = 53)	29.84	239.10 (*n* = 72)	15.80	0.21
GRO	1549.0 (*n* = 53)	139.50	1393.0 (*n* = 72)	95.70	0.34
Growth Factors					
EGF	158.70 (*n* = 53)	18.57	102.30 (n = 70)	8.17	0.003**
EFF-2	108.60 (*n* = 53)	9.57	79.30 (*n* = 72)	4.98	0.004**
FLT-3 L	11.62 (*n* = 20)	2.92	22.31 (*n* = 7)	7.87	0.12
G-CSF	39.72 (*n* = 52)	3.68	31.48 (*n* = 72)	2.19	0.04*
GM-CSF	11.77 (*n* = 30)	1.51	9.77 (*n* = 44)	1.14	0.29
TGF-α	2.02 (*n* = 21)	0.39	1.20 (*n* = 22)	0.20	0.07
VEGF	170.40 (*n* = 53)	16.72	133.10 (*n* = 71)	13.69	0.08
Platelet Marker					
sCD40L	1555.0 (*n* = 53)	256.90	967.80 (*n* = 72)	146.80	0.04*
Adipokine					
Adiponectin	4175.0 (*n* = 48)	572.30	5044.0 (*n* = 63)	400.20	0.20
Hepatokine					
Fetuin-A/AHSG	677.10 (*n* = 53)	51.21	612.30 (*n* = 72)	32.67	0.27

Note: Sample numbers <53 for diabetic and <72 for non-diabetic groups represent the missing data due to undetectable analyte levels in certain samples

*significant; **highly significant; ***extremely significant

**Table 3:** Correlation of plasma fetuin-A with inflammatory cytokines, chemokines and activation biomarkers in diabetic and non-diabetic individuals

Analyte	Diabetic		Non-diabetic	
	Pearson r	*P*	Pearson r	*P*
Cytokines				
IFN-α2	−0.44 (*n* = 52)	0.001^**^	0.04 (*n* = 56)	0.76
IFN-γ	−0.41 (*n* = 49)	0.003^**^	0.05 (*n* = 66)	0.68
IL-1α	−0.48 (*n* = 46)	0.001^**^	0.04 (*n* = 56)	0.76
IL-1β	−0.57 (*n* = 46)	<0.0001^***^	−0.33 (*n* = 58)	0.01^*^
IL-1RA	−0.46 (*n* = 52)	0.001^**^	0.24 (*n* = 63)	0.06
IL-3	−0.30 (*n* = 24)	0.16	−0.79 (*n* = 14)	0.001^**^
IL-4	−0.48 (*n* = 49)	0.001^**^	−0.01 (*n* = 60)	0.97
IL-5	−0.0008 (*n* = 20)	0.99	0.58 (*n* = 19)	0.01^*^
IL-6	−0.30 (*n* = 29)	0.12	0.08 (*n* = 33)	0.67
IL-7	−0.52 (*n* = 51)	<0.0001^***^	−0.38 (*n* = 61)	0.003^**^
IL-9	−0.47 (*n* = 18)	0.05^*^	−0.68 (*n* = 9)	0.04^*^
IL-10	−0.18 (*n* = 40)	0.26	0.11 (*n* = 36)	0.52
IL-12p40	−0.45 (*n* = 39)	0.004^**^	−0.23 (*n* = 36)	0.17
IL-12p70	−0.41 (*n* = 49)	0.003^**^	−0.01 (*n* = 59)	0.92
IL-13	−0.18 (*n* = 26)	0.39	0.56 (*n* = 20)	0.01^*^
IL-15	−0.52 (*n* = 41)	0.0005^***^	−0.49 (*n* = 34)	0.003^**^
IL-17A	−0.54 (*n* = 40)	0.0004^***^	−0.24 (*n* = 50)	0.09
TNF-α	−0.66 (*n* = 50)	<0.0001^***^	−0.33 (*n* = 72)	0.04^*^
TNF-β/ LT-α	−0.20 (*n* = 20)	0.46	0.62 (*n* = 16)	0.01^*^
Chemokines				
CCL-2/MCP-1	−0.69 (*n* = 53)	<0.0001^***^	−0.52 (*n* = 72)	<0.0001^***^
CCL-3/MIP1-α	0.01 (*n* = 48)	0.93	0.30 (*n* = 53)	0.03^*^
CCL-4/MIP1-β	−0.38 (*n* = 53)	0.005^**^	0.20 (*n* = 71)	0.10
CCL-5/RANTES	0.04 (*n* = 48)	0.80	0.30 (*n* = 63)	0.02^*^
CCL-7/MCP-3	0.16 (*n* = 10)	0.67	0.80 (*n* = 14)	<0.0001^***^
CCL-11/Eotaxin-1	−0.42 (*n* = 53)	0.002^**^	−0.19 (*n* = 72)	0.10
CCL-22/MDC	−0.47 (*n* = 53)	0.0003^***^	−0.32 (*n* = 72)	0.006^**^
CX3CL-1/Fractalkine	−0.56 (*n* = 53)	<0.0001^***^	−0.30 (*n* = 70)	0.01^*^
CXCL-8/IL-8	−0.52 (*n* = 47)	<0.0001^***^	0.24 (*n* = 52)	0.09
CXCL-10/IP-10	−0.22 (*n* = 53)	0.12	−0.50 (*n* = 72)	<0.0001^***^
Growth-related oncogene (GRO)	−0.69 (*n* = 53)	<0.0001^***^	−0.29 (*n* = 72)	0.01^*^
Adipokine				
Adiponectin	−0.14 (*n* = 48)	0.34	−0.01 (*n* = 63)	0.94
Growth Regulatory Factors				
EGF	−0.42 (*n* = 53)	0.002^**^	−0.03 (*n* = 70)	0.84
EFF-2	−0.61 (*n* = 53)	<0.0001^***^	−0.29 (*n* = 72)	0.01^*^
FLT-3 L	−0.32 (*n* = 20)	0.16	−0.08 (*n* = 7)	0.87
G-CSF	−0.42 (*n* = 52)	0.002^**^	−0.02 (*n* = 72)	0.89
GM-CSF	−0.45 (*n* = 30)	0.01^*^	0.82 (*n* = 44)	0.06
TGF-α	0.03 (*n* = 21)	0.89	0.36 (*n* = 22)	0.10
VEGF	−0.53 (*n* = 53)	<0.0001^***^	−0.29 (*n* = 71)	0.01^*^
Platelet Activation Marker				
sCD40L	−0.64 (*n* = 53)	<0.0001^***^	−0.48 (*n* = 72)	<0.0001^***^

Note: Sample numbers <53 for diabetic and <72 for non-diabetic groups represent the missing data due to undetectable analyte levels in certain samples

*significant; **highly significant; ***extremely significant

### Inflammatory cytokines suppress fetuin-A production in HepG2 cell cultures

**Fig. 1:**
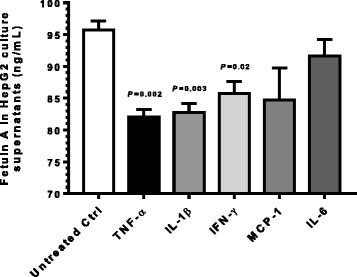
Fetuin A in supernatants of HepG2 cell cultures treated with proinflammatory cytokines. Human hepatocellular carcinoma HepG2 cells were cultured at 37 °C with 5 % CO_2_ in high-glucose DMEM medium containing 10 % fetal bovine serum, 100 units/mL penicillin, and 100 μg/mL streptomycin in 6-well plates at a density of 0.25 × 10^6^ cells/mL until about 70 % confluence and old medium was replaced with fresh medium. Cell monolayers were then treated with rhTNF-α (50 ng/mL), rhIL-1β (10 ng/mL), rhIFN-γ (50 ng/mL), rhMCP-1 (40 ng/mL), and rhIL-6 (100 ng/mL) and incubated at 37 °C for 24 h. Cell supernatants were collected and fetuin-A levels were measured using sandwich high-sensitivity ELISA (Human fetuin-A PicoKine^TM^ ELISA kit, Boster Biological Technology, USA) following the manufacturer’s instructions as described in Patients and Methods. Fetuin-A production (mean ± SEM) was found to be significantly suppressed in HepG2 cells treated with TNF-α (82.05 ± 1.16 ng/mL, *P* = 0.002), IL-1β (82.73 ± 1.45 ng/mL, *P* = 0.003), and IFN-γ (85.70 ± 1.93 ng/mL, *P* = 0.02) as compared with untreated control (95.73 ± 1.43 ng/mL). However, fetuin-A production in cells treated with MCP-1 (84.74 ± 5.02 ng/mL, *P* = 0.10) and IL-6 (91.66 ± 2.55 ng/mL, *P* = 0.24) differed non-sidnificantly from control. The representative data from three independent determinations are shown

Since metformin and angiotensin receptors blockers have been reported to have lowering effects on circulating fetuin-A levels [7–9], therefore, possible confounding (suppressive) effects of therapy in our study cohort may not be ruled out. We asked if the signature inflammatory cytokines, related with T2D pathogenesis, alone were able to suppress the production of fetuin-A in an in vitro HepG2 cell culture model. To address this, we treated HepG2 cell cultures (at 70–80 % confluence) with the typical proinflammatory cytokines and chemokine that are known to be upregulated in metabolic disease such as obesity and T2D and measured the expression of fetuin-A in supernatants for comparison with untreated controls. The treatments with these cytokines/chemokine were carried out using standard concentrations as available from the literature and these cytokines/chemokine concentrations also activated the respective signaling pathways in HepG2 cells (data not shown). Our data show that fetuin-A production was significantly reduced (*P* ≤ 0.05) in HepG2 cell cultures at 24 h following treatments with TNF-α, IL-1β, and IFN-γ while a non-significant suppression (*P* > 0.05) was observed after HepG2 cell treatment with MCP-1 and IL-6 as compared with controls (Fig. 1). In addition, time course experiments were also performed in which both the proinflammatory (IFN-γ and TNF-α) and antiinflammatory (IL-4 and IL-10) cytokines were used to treat HepG2 cells and fetuin-A was measured in culture supernatants at 1, 6, 12, 24, and 48 h. These data show (Additional file 1: Figure S1) that TNF-α treatment resulted in fetuin-A suppression at as early as 12 h and both IFN-γ and TNF-α suppressed fetuin-A at 24 h and 48 h while on the other hand, IL-10 induced the expression of fetuin A at these time points. As expected, no differences among treatments were observed at earlier (1 h and 6 h) time points since they may not represent the optimal time period required for fetuin-A synthesis and extracellular expression following the cytokine treatments used.

## Discussion

Fetuin-A is a relatively new hepatokine and its significance as a predictive biomarker in the T2D-associated metabolic inflammation remains unclear. We report, for the first time to our knowledge, that the plasma fetuin-A levels correlate negatively with those of proinflammatory cytokines/chemokines and activation biomarkers in T2D individuals which represents the predictive significance of fetuin-A as a negative APP in T2D. Several lines of evidence from the previous studies suggest that fetuin-A synthesis can be divergently regulated in various disease conditions and thus this hepatokine can serve as a positive or negative APP during the morbid states. The elevated levels of fetuin-A as found in trauma and ischemic injury or stroke suggest its role as a positive APP in those disease conditions [10, 11] whereas, the reduced fetuin-A levels as observed in endotoxemia, sepsis, and other inflammatory conditions including pancreatitis, chronic kidney disease, and rheumatoid arthritis point to its antiinflammatory or protective role as a negative APP during these morbid states [12–15]. Our data reveal a strong negative association between fetuin-A levels and those of signature inflammatory mediators or activation markers such as TNF-α, IL-1β, IL-7, IL-15, CCL-2, CCL-4, CXCL-8, CX3CL-1, EFF2, GRO, sCD40L, and VEGF in T2D patients. The negative association between fetuin-A and inflammatory mediators shown by our clinical data is further supported by our in vitro studies indicating the reduced fetuin-A production in HepG2 cells treated with proinflammatory cytokines such as TNF-α, IL-1β, and IFN-γ. In agreement with our results, early-stage proinflammatory cytokines such as TNF-α, IL-1β and IFN-γ were reported to act as negative regulators of fetuin-A synthesis [12]. The inverse relationship that we found between fetuin-A levels and proinflammatory cytokines/chemokines in T2D patients implies that one would expect to find lower fetuin-A levels in the circulation in the presence of increased proinflammatory cytokines/chemokines and, hence, represent a poor disease prognosis in inflammatory states. This line of argument is supported by Metry et al. study showing that low serum fetuin-A levels were the predictor of a poor outcome in hemodialysis patients with inflammation [15]. The protective role of fetuin-A as a negative APP is also substantiated by its function as an opsonin in macrophage deactivation at sites of inflammation [16]. On the other hand, abnormally elevated fetuin-A *per se* may also play a pathogenic role by inducing defective insulin receptor signaling, TLR-4 activation, macrophage migration, adipocyte dysfunction, hepatic inflammation, and fibrosis [12, 17].

Our clinical data further show that plasma fetuin-A levels were associated positively with those of certain T_H_2 cytokines (IL-5, IL-13) and C-C chemokines (CCL-3, CCL-5, and CCL-7); however, only in non-diabetic individuals. It is not yet clear if these cytokines/chemokines are the positive regulators of fetuin-A synthesis, as for instance HMGB1 which is a late-acting proinflammatory mediator and was shown to induce fetuin-A in systemic inflammatory conditions [12–15]. In addition, Polyzos et al. commented on the potentially dual faceted nature of fetuin-A in *Helicobacter pylori* infection and insulin resistance [18]. Trepanowski et al. reported that circulating fetuin-A levels were elevated in conditions like obesity, T2D, non-alcoholic fatty liver disease, and metabolic syndrome while the changes associated with impaired insulin sensitivity and glucose tolerance [19]. Interestingly, two other studies examined the association between circulating fetuin-A and prevalent peripheral arterial disease in T2D patients; however, one study reported a positive association [5] and the other study reported a negative association [20]. Such discrepancies may relate to the presence of complex confounding factors in T2D patients such as immunometabolic differences, co-morbidities, and widely varying therapeutic regimens. Our study further reveal no association between plasma fetuin-A levels and those of IL-6 (revealed by both in vivo and in vitro data), IL-10, adiponectin, FLT-3 L, and TGF-α in diabetic and non-diabetic individuals. IL-6 is regarded as a pleiotropic cytokine which may have both inflammatory and antiinflammatory effects, IL-10 is a well-known antiinflammatory cytokine, adiponectin is an adipokine that suppresses metabolic derangements associated with obesity/T2D, FLT-3 L is a growth factor for hematopoietic progenitors, and TGF-α is a mitogenic polypeptide that acts as a ligand for EGFR/HER-1 to activate signaling pathways for cell proliferation, differentiation, and development [21–23]. Thus, fetuin-A may not be a sensitive predictor to evaluate changes in the pleiotropic and antiinflammatory cytokines as well as certain adipokines or growth factors involved in metabolic disease.

Notably, in our study, fetuin-A levels were found to be comparable between diabetic and non-diabetic individuals whereas those of proinflammatory cytokine/chemokines or activation/growth mediators (IL-1α/β, TNF-α, IFN-α2, IL-12, MCP-1, RANTES, eotaxin-1, fractalkine, EGF, EFF-2, sCD40L, G-CSF, etc.) were found to be significantly higher in diabetic as compared with non-diabetic individuals which counteracts the argument of a positive association between fetuin-A and these inflammatory mediators. Our in vitro data further support the negative association between fetuin-A and signature inflammatory cytokines such as TNF-α, IL-1-β, and IFN-γ and thus rule out the possible fetuin-A suppressive effects of the antidiabetic and antihypertensive therapy in T2D patients. The time course experiments further validate these data and show that optimal effects of cytokine treatments in HepG2 cells were induced at 24 h and 48 h whereas TNF-α-mediated suppression of fetuin-A was observed at as early as 12 h. Interestingly, IL-10 rather upregulated the expression of fetuin-A in HepG2 cells at 24 h and 48 h time points.

## Conclusions

Taken together, our data show that plasma fetuin-A levels correlated negatively with inflammatory cytokines/chemokines and various activation biomarkers in T2D patients, indicating that the circulatory fetuin-A may have predictive importance as a negative APP in metabolic disease. Nonetheless, further studies will be required to validate these data in larger patient cohorts.

## Additional file

Additional file 1: Figure S1. Fetuin A in supernatants of HepG2 cell cultures pretreated with proinflammatory and antiinflammatory cytokines. HepG2 cells were cultured at 37 °C with 5 % CO_2_ in high-glucose DMEM medium containing 10 % fetal bovine serum, 100 units/mL penicillin, and 100 μg/mL streptomycin in 6-well plates at a density of 0.25 × 10^6^ cells/mL until about 70 % confluence was reached and old medium was replaced with fresh medium. Cell monolayers (triplicate wells) were treated with proinflammatory cytokines, such as rhIFN-γ (50 ng/mL) and rhTNF-α (50 ng/mL) as well as antiinflammatory cytokines, such as rhIL-4 (20 ng/mL), and rhIL-10 (30 ng/mL) and cultures were incubated at 37 °C for 1 h, 6 h, 12 h, 24 h, and 48 h for time course analysis. Cell supernatants were collected and fetuin-A levels were measured using sandwich high-sensitivity ELISA (Human fetuin-A PicoKine^TM^ ELISA kit, Boster Biological Technology, USA) following the manufacturer’s instructions as described in Patients and Methods. Fetuin A levels differed non-significantly at 1 h (A) and 6 h (B) while TNF-α induced significant suppression at 12 h (175.4 ± 2.5 ng/mL, *P* = 0.04) as compared with controls (185.7 ± 1.7 ng/mL) (C). At 24 h post-treatment, fetuin-A levels (mean ± SEM) were found to be significantly suppressed in HepG2 cells treated with IFN-γ (149.1 ± 0.5 ng/mL, *P* = 0.04) and TNF-α (149.6 ± 1.4 ng/mL, *P* = 0.05) as compared with untreated controls (156.8 ± 1.7 ng/mL) (D). Similarly at 48 h, fetuin-A expression was found to be significantly reduced following treatments with IFN-γ (122.5 ± 2.2 ng/mL, *P* = 0.02) and TNF-α (126.0 ± 1.7 ng/mL, *P* = 0.04) as compared with untreated controls (133.8 ± 1.5 ng/mL) (E). On the other hand, treatment with IL-10 upregulated the fetuin-A expression at both 24 h (164.0 ± 0.5 ng/mL, *P* = 0.01) and 48 h (143.0 ± 1.2 ng/mL, *P* = 0.01) as compared with respective controls. The representative data from three independent determinations are shown. (PDF 93 kb)

## Abbreviations

ABC: Avidin-Biotin-Peroxidase Complex
AHSG: α-2 Heremans-Schmid glycoprotein
APP: Acute phase protein
BMI: Body mass index
CCL: C-C motif ligand
CXCL: C-X-C motif ligand
EFF: Elongation factor F
EGF: Epidermal growth factor
FLT-3 L: FMS-like tyrosine kinase 3 ligand
G-CSF: Granulocyte-colony stimulating factor
GM-CSF: Granulocyte-macrophage colony stimulating factor
GRO: Growth-related oncogene
HbA1c: Glycated hemoglobin
IFN: Interferon
IL: Interleukin
LT: Lymphotoxin
MCP-1: Macrophage chemoattractant protein-1
MDC: Macrophage-derived chemokine
MFI: Mean fluorescence intensity
MIP: Macrophage inflammatory protein
OD: Optical density
OGTT: Oral glucose tolerance test
sCD40L: soluble CD40 ligand
T2D: Type-2 diabetes
TBS: Tris-buffered saline
TGF: Transforming growth factor
TLR: Toll-like receptor
TMB: 3,3',5,5'-Tetramethylbenzidine
TNF: Tumor necrosis factor
VEGF: Vascular endothelial growth factor
