# Supplementary figures and images for: Plasma fetuin-A/α2-HS-glycoprotein correlates negatively with inflammatory cytokines, chemokines and activation biomarkers in individuals with type-2 diabetes
Source: BMC Immunol. 2016 Sep 26;17:33. doi: 10.1186/s12865-016-0171-y (PMC5037637; doi:10.1186/s12865-016-0171-y)

**A**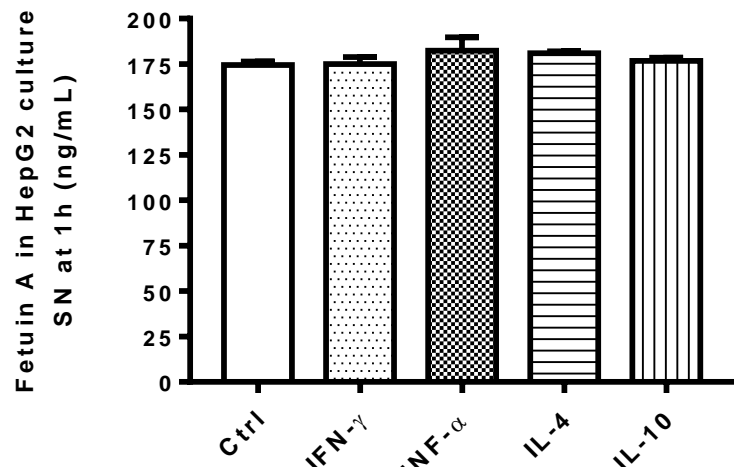**B**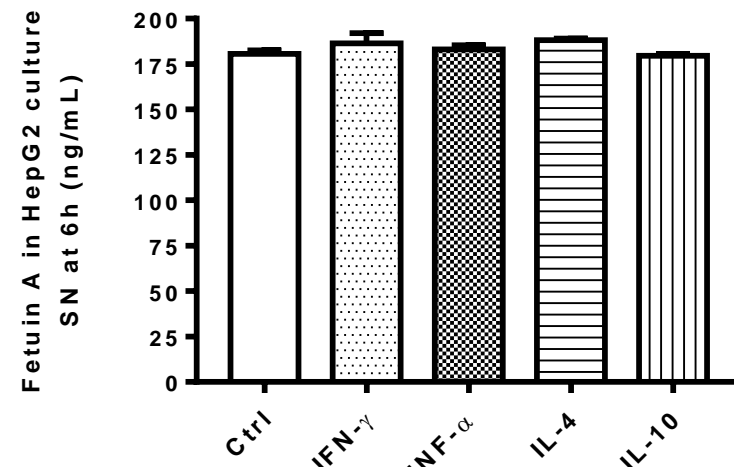**C**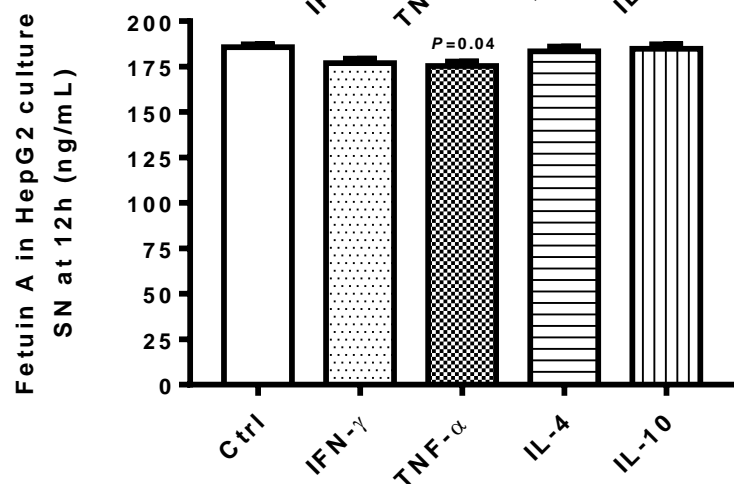**D**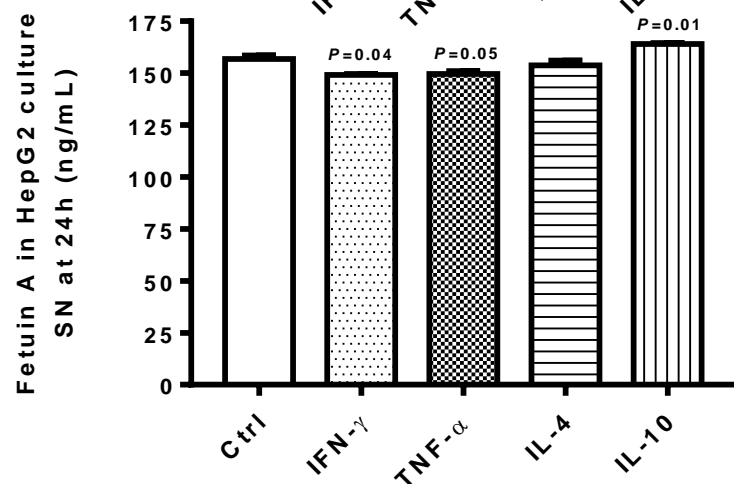**E**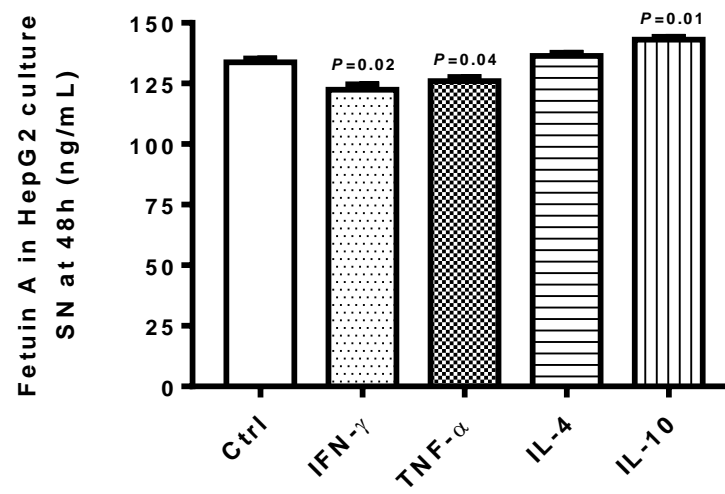**Figure S1**

Supplement: Additional file 1: Figure S1. — Fetuin A in supernatants of HepG2 cell cultures pretreated with proinflammatory and antiinflammatory cytokines. HepG2 cells were cultured at 37 °C with 5 % CO2 in high-glucose DMEM medium containing 10 % fetal bovine serum, 100 units/mL penicillin, and 100 μg/mL streptomycin in 6-well plates at a density of 0.25 × 10^6^ cells/mL until about 70 % confluence was reached and old medium was replaced with fresh medium. Cell monolayers (triplicate wells) were treated with proinflammatory cytokines, such as rhIFN-γ (50 ng/mL) and rhTNF-α (50 ng/mL) as well as antiinflammatory cytokines, such as rhIL-4 (20 ng/mL), and rhIL-10 (30 ng/mL) and cultures were incubated at 37 °C for 1 h, 6 h, 12 h, 24 h, and 48 h for time course analysis. Cell supernatants were collected and fetuin-A levels were measured using sandwich high-sensitivity ELISA (Human fetuin-A PicoKineTM ELISA kit, Boster Biological Technology, USA) following the manufacturer’s instructions as described in Patients and Methods. Fetuin A levels differed non-significantly at 1 h (A) and 6 h (B) while TNF-α induced significant suppression at 12 h (175.4 ± 2.5 ng/mL, P = 0.04) as compared with controls (185.7 ± 1.7 ng/mL) (C). At 24 h post-treatment, fetuin-A levels (mean ± SEM) were found to be significantly suppressed in HepG2 cells treated with IFN-γ (149.1 ± 0.5 ng/mL, P = 0.04) and TNF-α (149.6 ± 1.4 ng/mL, P = 0.05) as compared with untreated controls (156.8 ± 1.7 ng/mL) (D). Similarly at 48 h, fetuin-A expression was found to be significantly reduced following treatments with IFN-γ (122.5 ± 2.2 ng/mL, P = 0.02) and TNF-α (126.0 ± 1.7 ng/mL, P = 0.04) as compared with untreated controls (133.8 ± 1.5 ng/mL) (E). On the other hand, treatment with IL-10 upregulated the fetuin-A expression at both 24 h (164.0 ± 0.5 ng/mL, P = 0.01) and 48 h (143.0 ± 1.2 ng/mL, P = 0.01) as compared with respective controls. The representative data from three independent determinations are shown. (PDF 93 kb) [file 12865_2016_171_MOESM1_ESM.pdf]
